# Effects of *Bacillus subtilis KG109* on Growth Performance, Carcass Quality, Serum Indicators, Intestinal Morphology, and Digestive Enzymes in Broilers

**DOI:** 10.3390/ani14243650

**Published:** 2024-12-18

**Authors:** Hong Chen, Weixin Liu, Hao Zhang, Yibo Yan, Meiqi Chen, Xiaoling Ding, Cheng Zhang, Runsheng Jiang, Zaigui Wang

**Affiliations:** 1 College of Animal Science and Technology, Anhui Agricultural University, Hefei 230031, China; 23720314@stu.ahau.edu.cn (H.C.); 22720181@stu.ahau.edu.cn (W.L.); 23720311@stu.ahau.edu.cn (H.Z.); 18315579471@163.com (Y.Y.); 13303859811@163.com (M.C.); cheng20050502@126.com (C.Z.); jiangrunshen@ahau.edu.cn (R.J.); 2 College of Life Science and Technology, Anhui Agricultural University, Hefei 230031, China; wangzaigui2013@163.com

**Keywords:** *Bacillus subtilis*, broiler, growth performance, carcass quality, serum biochemical indexes, serum antioxidants, immune indexes, intestinal morphology, intestinal enzyme activities

## Abstract

The purpose of this experiment is to investigate how different doses of *Bacillus subtilis KG109* powder affect the growth performance, carcass quality, serum biochemical indexes, serum antioxidant and immunological index, intestinal morphology, and digestive enzyme activity of broilers. Four hundred chicks of a similar weight (1 day old) are randomly assigned to four groups of five replicates of 20 chicks each (half males and half females). The control group is fed a basal ration, and the experimental groups T1, T2, and T3 are supplemented with 6.0 × 10^8^ CFU/kg, 1.2 × 10^9^ CFU/kg, and 1.8 × 10^9^ CFU/kg of *Bacillus subtilis KG109* bacterial powder, respectively, in the basal ration. The feeding cycle is 52 d. Compared with the control group, *Bacillus subtilis KG109* powder (1) increases the broiler feed conversion ratio (FCR) (*p* < 0.05), (2) improves the carcass quality (slaughter rate, cooking loss, L* and b* values) (*p* < 0.05), (3) enhances the serum biochemical indexes (alanine transaminase (ALT), alkaline phosphatase (ALP), aspartate transaminase (AST), albumin (ALB), and triglycerides (TG)) (*p* < 0.05), (4) improves the serum antioxidant capacity (total an-tioxidant capacity (T-AOC), total superoxide dismutase (T-SOD), and glutathione peroxidase (GSH-PX)) and immunoglobulins (lg A, lg G, lg M) (*p* < 0.05), (5) improves the intestinal morphology (villus height and villus height to crypt depth (VCR)) (*p* < 0.05), and (6) increases the intestinal digestive enzyme activities (amylase, protease, and lipase) (*p* < 0.05). In summary, adding *Bacillus subtilis KG109* to broiler diets can result in a significant decrease in broilers’ FCR, an increase in their slaughtering rate, a decrease in their serum ALT, ALP, and AST activities, an increase in their serum TG content, an improvement of their immune and antioxidant capacity, an improvement of their intestinal morphology, and an improvement of their intestinal digestive enzyme activity. It is recommended to add 1.8 × 10^9^ CFU/kg of bacteria.

## Simple Summary

*Bacillus subtilis* is frequently utilized as a ration addition in the livestock and poultry industries; however, different strains of *Bacillus subtilis*, dosages, and subjects of research yield diverse outcomes. Therefore, this study shows that the addition of 6.0 × 10^8^ CFU/kg, 1.2 × 10^9^ CFU/kg, and 1.8 × 10^9^ CFU/kg of *Bacillus subtilis KG109* powder to diets improves growth performance, carcass quality, serum indicators, intestinal morphology, and digestive enzyme activities in broilers. The study finds that *Bacillus subtilis KG109* considerably increases broiler chicken performance, with 1.8 × 10^9^ CFU/kg being the best dosage of diet supplementation.

## 1. Introduction

Probiotics have become one of the ideal alternatives to antibiotics in animal husbandry, with probiotics emerging as an effective alternative to antibiotics in the livestock business. Probiotics differ from antibiotics because of their natural environmental safety, the absence of toxic residues in eggs and meat, the promotion of healthy gut flora, and enhanced feed intake, feed conversion, and growth rates [1]. Currently, *Bacillus subtilis* is one of the most widely used probiotics in poultry feed [2] owing to its remarkable resilience and durability in both feed processing and the broilers’ gastrointestinal tract, making it an excellent contender for improving the health of chickens [3]. Previous research has shown that *Bacillus subtilis* has high anti-inflammatory and antibacterial capabilities [4] and a positive effect on promoting the growth of livestock and poultry [5,6], improving carcass quality [7,8], improving serum biochemical indexes [6], enhancing serum antioxidant as well as immune abilities [9], improving intestinal morphology [10], improving the activity of intestinal-related enzymes [11], and maintaining the balance of the intestinal flora [12].

As a result, *Bacillus subtilis* is utilized as a premium feed supplement in broiler diets; however, an increasing number of studies have discovered that the effects of different strains and dosages of *Bacillus subtilis* vary. For instance, broilers have been shown to be affected differently by the addition of 5.0 × 10^8^ CFU/kg of *Bacillus subtilis ATCC19659* [13], 1.0 × 10^9^ CFU/kg of *Bacillus subtilis DSM32315* [14], 5.0 × 10^8^ CFU/kg of *Bacillus subtilis HC6* [9], 5.0 × 10^8^ CFU/kg of *Bacillus subtilis DSM29784* [15], and 4.0 × 10^10^ CFU/kg of *Bacillus subtilis fmbj* [16]. This could be because the efficacy of probiotics varies depending on an array of variables, including the kind, strain, mode of administration, dosage, and duration of usage [17]

In this study, *Bacillus subtilis KG109* is chosen as the research object to study its effect on broiler chickens, as its isolation in our laboratory fermented feed has been found to significantly increase the small peptide, protein, and fat content of the feed while significantly reducing the fiber content. Probiotics are available in a variety of forms for poultry use, such as drops, sprays, granules, tablets, coated capsules, or sachets of powder [18]. As a result, this study investigates the addition of *Bacillus subtilis KG109* powder to broiler feed rations, and holistically studies the effects of adding different doses of *Bacillus subtilis KG109* powder to feed rations on broiler growth performance, carcass quality, serum biochemical indexes, serum antioxidant and immune indexes, intestinal morphology, and digestive enzyme activities, providing guidance for the rational use of this strain.

## 2. Materials and Methods

### 2.1. Materials

*Bacillus subtilis KG109* was isolated from laboratory-fermented feeds and cultured on a Luria–Bertani medium (LB) to selectively grow spore-forming bacteria. According to the method of strain identification by Gadde et al. [1], the cultivated spore-forming bacteria were identified as *Bacillus subtilis*, with *Bacillus subtilis KG109* transformed into *Bacillus subtilis* powder through strain activation, cultivation, drying, etc. The *Bacillus subtilis KG109* powder was sprinkled uniformly over the basal diet in different doses and the feed was then mixed thoroughly (the different doses were: (T1) 6.0 × 10^8^ CFU/kg, (T2) 1.2 × 10^9^ CFU/kg, and (T3) 1.8 × 10^9^ CFU/kg). The testing period was from 1 d to 52 d of age. Two feeding phase diets were utilized: a starter diet from days 1 to 21 and a grower diet from days 22 to 52. The diets were formulated to meet the nutrient requirements recommended by the NRC (1994). The nutritional makeup is indicated in Table 1.

### 2.2. Methods

The experiment comprised 400 1-day-old 817 hybrid broilers (817 is a tiny broiler breed with local features in China) of similar body weights. They were randomly divided into four groups containing five replicates each, each with 20 chicks (1/2 males and 1/2 females, in separate cages). The broilers were separately fed (1) the basal ration for 52 d (CON), (2) the basal diet supplemented with 6.0 × 10^8^ CFU/kg *Bacillus subtilis* for 52 d (treatment1, T1), (3) the basal diet supplemented with 1.2 × 10^9^ CFU/kg *Bacillus subtilis* for 52 d (treatment, T2), and (4) the basal diet supplemented with1.8 × 10^9^ CFU/kg *Bacillus subtilis* for 52 d (treatment3, T3). The experiment lasted for 52 days. The feeding trials took place at the Muzi Poultry Cooperative in Xuancheng City, Anhui Province, in cages with natural ventilation in the chicken house. Daylight was eliminated during this study, and 18 h of lighting was provided from incandescent bulbs. During the test period, all chickens were fed and hydrated ad libitum, relative humidity was maintained between 55% and 65%, routine disinfection was performed on a regular basis, and vaccination was performed in accordance with broiler chicken rearing methods.

### 2.3. Sample Collection and Indicator Measurement

#### 2.3.1. Growth Performance

During the experimental period of 1–52 d, the feed intake of each replicate was recorded daily, and the broilers were weighed at the mid-term, at 21 days of age; 1 day before the end of the experiment, the broilers were fasted for 12 h and weighed in the morning of the following day. At the end of the experiment, the average daily feed intake (ADFI), the average daily gain (ADG), and the feed conversion ratio (FCR) were calculated for each group of chickens during the experimental period.

#### 2.3.2. Carcass Quality

At the end of the 52 d experiment, 10 chickens (two per replicate, one male and one female) close to the mean body weight were randomly chosen from each group, fasted and not watered, and slaughtered after 12 h. Weights for the carcass, complete clean rump, semi-clean rump, breast muscle, leg muscle, and abdominal fat were all weighed. Each chicken’s left breast muscle was also tested for pH_1h_, drip loss, cooking loss, softness, lightness (L*), redness (a*), and yellowness (b*).

#### 2.3.3. Serum Biochemical Indexes

At the end of the 52 d experiment, ten chickens (two per replicate, one male and one female) with a near-average body weight were randomly chosen from each group and fasted for 12 d of sample collection. A non-anticoagulant vacuum blood collection tube was used to collect approximately 10 mL of blood from the wing vein. The blood was then centrifuged at 3500 r/min for 10 min and stored at −20 °C for measurement. The Mindray BS-220 automatic biochemistry analyzer was used to measure serum alanine transaminase (ALT), aspartate transaminase (AST), alkaline phosphatase (ALP) activity, total protein (TP), albumin (ALB), globulin (GLB), uric acid (UA), total cholesterol (TC), and triglycerides (TG). The white globule ratio (albumin/globulin, A/G) was calculated.

#### 2.3.4. Determination of Serum Antioxidant Indexes and Immunological Indexes

The serum was collected using the method described in Section 2.3.3. Serum total superoxide dismutase (T-SOD), glutathione peroxidase (GSH-PX) activity, total antioxidant capacity (T-AOC), malondialdehyde (MDA), immunoglobulin G (IgG), immunoglobulin M (IgM), and immunoglobulin A (IgA) were measured using the enzyme-linked immunosorbent assay (ELISA), with kits purchased from Wuhan Xinqidi Biotechnology Co., Wuhan, China. (Refer to Appendix A for details about the kit.)

#### 2.3.5. Intestinal Morphology and Intestinal Digestive Enzyme Activity

As Section 2.3.2, the broiler chickens were slaughtered, a total of 5 cm of duodenum and jejunum intestinal mesenteries in the small intestine was clipped from each, the intestinal lumen was rinsed in saline and then fixed in 10% formaldehyde in preparation for paraffin sections, and the slides were stained with traditional Meyer Hematoxylin and Eosin [19] and then examined under a laser scanning confocal microscope. The image processing and analysis software Image-Pro Plus version 6 (Rockville, MD, USA) was used to measure the intestinal villus height (VH), crypt depth (CD), and villus-to-crypt ratio (VCR).

The contents of the duodenum and jejunum were collected into freezing tubes and stored at −80 °C. The activity of protease, amylase, and lipase was determined using enzyme-linked immunosorbent assay (ELISA) kits purchased from Wuhan Xinqidi Biotechnology Co. (Refer to Appendix A for details about the kit).

### 2.4. Statistics and Analysis of Data

The SPSS 20.0 statistical software was used to perform one-way ANOVA tests on the experimental results. The Duncan’s test was conducted for multiple comparisons, with *p* < 0.05 indicating a significant difference, *p* < 0.01 indicating a highly significant difference, and *p* > 0.05 indicating an insignificant difference. The results are expressed as mean ± standard error.

## 3. Results

### 3.1. Effect of Dietary Supplementation with Bacillus subtilis KG109 on the Growth Performance of Broiler Chickens

Table 2 shows that FCR was significantly lower in the T3 group compared to the CON group from days 1 to 21 (*p* < 0.05), in the T2 and T3 groups compared to the CON group from days 22 to 52 (*p* < 0.05 or *p* < 0.01), and in the T1, T2, and T3 groups compared to the CON group from days 1 to 52 (*p* < 0.05 or *p* < 0.01).

### 3.2. Effect of Dietary Supplementation with Bacillus subtilis KG109 on the Carcass Quality of Broiler Chickens

Table 3 shows that the broilers in the T2 and T3 groups had significantly greater slaughter rates (*p* < 0.05 or *p* < 0.01) compared to the CON group. Table 4 shows that the cooking loss of broiler breast muscle in the T1 and T2 groups was significantly or highly significantly lower than that in the CON group (*p* < 0.05 or *p* < 0.01), the L* value of the broiler breast muscle in the T2 group was significantly higher than that in the CON group (*p* < 0.05), and the b* value of the broiler breast muscle in the experimental groups was highly significantly lower than that in the CON group (*p* < 0.01), while the differences of all other indexes were not significant.

### 3.3. Effect of Dietary Supplementation with Bacillus subtilis KG109 on the Serum Biochemical Indexes of Broiler Chickens

As shown in Table 5, the serum ALT and ALP activity of the broilers in the test groups was significantly higher than that in the CON group (*p* < 0.05), the serum AST activity of the broilers in the T3 group was significantly higher than that in the T1 group (*p* < 0.05), the serum ALB content of the broilers in the T2 and T3 groups was significantly higher than that in the CON group (*p* < 0.05), and the serum TG content of the broilers in the T3 group was extremely significantly lower than that in the CON and T2 groups (*p* < 0.01).

### 3.4. Effect of Dietary Supplementation with Bacillus subtilis KG109 on the Serum Antioxidant and Immune Indexes of Broiler Chickens

Table 6 shows that broilers in the test groups had significantly higher serum T-SOD levels, GSH-Px activity, and T-AOC levels than those in the CON group (*p* < 0.01), serum MDA levels were significantly lower in the T2 and T3 groups compared to the CON group (*p* < 0.01), and serum IgG, IgM, and IgA levels were significantly higher in the test group compared to the CON group (*p* < 0.01).

### 3.5. Effect of Dietary Supplementation with Bacillus subtilis KG109 on the Intestinal Morphology of the Duodenum and Jejunum and on the Digestive Enzyme Activity of Broiler Chickens

**Figure 1:**
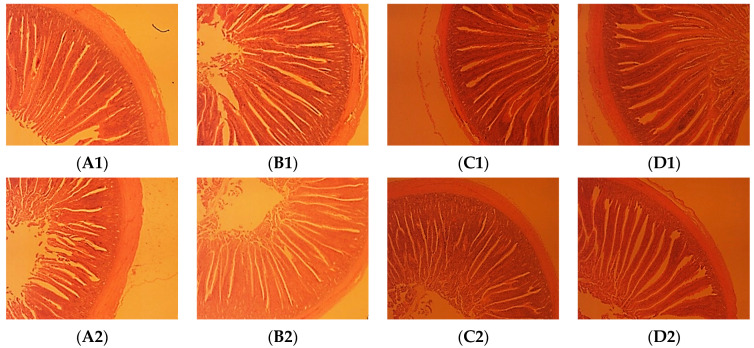
Slice atlases of duodenum and jejunum (40×). (**A1**–**D1**) represent the duodenum in the control, T1, T2, and T3 groups, respectively; (**A2**–**D2**) represent the jejunum in the control, T1, T2, T3 groups, respectively.

Table 7 shows that broilers in the test groups had significantly greater duodenal villus heights than those recorded in the CON group (*p* < 0.01), the broilers in the T1, T2, and T3 groups exhibited significantly higher duodenal VCR than those in the CON group (*p* < 0.05 or *p* < 0.01), and the broilers in the T2 and T3 groups had significantly greater jejunal villus heights than those recorded in the CON group (*p* < 0.05). Figure 1 shows how the intestinal morphology of the duodenum and jejunum of the broilers can be more intuitively observed by using paraffin.

Table 8 shows that the broilers in the test groups had significantly higher duodenal protease, amylase, and lipase activities compared to the CON group (*p* < 0.01), as well as significantly higher jejunal protease and amylase activities (*p* < 0.01) and jejunal lipase activities in the T2 and T3 groups compared to the CON group.

## 4. Discussion

### 4.1. Effect of Dietary Supplementation with Bacillus subtilis KG109 on the Growth Performance of Broiler Chickens

*Bacillus subtilis* is a probiotic that is commonly used in poultry feed production. Numerous studies have demonstrated that *Bacillus subtilis* can boost broiler chicken growth. The present study found that, compared to the control group, dietary supplementation with *Bacillus subtilis KG109* had a propensity to increase, although not at a statistically significant level, ADG and ADFI and caused a significant decrease in FCR in all experimental groups. Mardanova et al. [20] documented improved broiler body weights after supplementation with *Bacillus subtilis* in the diets. It has also been demonstrated that adding *Bacillus subtilis* to the ration has no significant influence on broiler ADG and ADFI, although it considerably lowers FCR and mortality while improving feed conversion [9,15]. These are in general agreement with the results of this study. Following the addition of the different concentrations used in this study, the FCR continued to decrease with increasing doses, but the effect of higher doses on FCR warrants further investigation.

### 4.2. Effect of Different Ratios of Bacillus subtilis Added to Diets on the Carcass Quality of Broiler Chickens

Slaughtering performance is one of the most important markers of broiler carcass quality, since it can visibly reflect the animal body composition and the proportion of edible components, and it is positively connected with carcass weight. The results of this experiment show that the slaughtering rate of the test groups increased significantly with increasing *Bacillus subtilis* doses in the diet compared to the control group, but the other indexes were not significant. It has been shown that the addition of 3. 0 × 10^10^ CFU/kg of *Bacillus subtilis* to the basal diet can significantly increase the carcass weight, half-clearance weight, and carcass rate of broilers, while improving the carcass quality of broilers [21]. The results are consistent with those derived from the present experiment.

Water-holding capacity is another essential indicator for evaluating carcass quality. Water-holding capacity, including drip loss and cooking loss, is associated with nutrition, flavor, and juiciness [22]. Lower drip and cooking losses suggest a greater water-holding capacity and a superior meat quality. The findings of this experiment reveal that adding *Bacillus subtilis KG109* to the rations had a tendency to reduce drip loss and cooking loss in all experimental groups compared to the control group, with the effect on cooking loss being significant. Wang et al. [23] and Tang et al. [14] show that the addition of *Bacillus subtilis* to the diets significantly reduce the drip loss and cooking loss of chicken meat, while increasing the water-holding capacity of the meat. These are consistent with the results of this experiment, in which the cooking loss of the broiler breast muscle in both T1 and T2 groups was significantly lower than that of the control group, with the T2 group reaching a highly significant level. The difference in the dripping loss of the breast muscle was not significant among groups, indicating that the addition of 1. 2 × 10^9^ CFU/kg *Bacillus subtilis* in the diet had the best effect on the cooking loss of the breast muscle.

Meat color is the most important organoleptic indicator, reflecting the brightness of the color of the muscle cross-section. The a*, b*, and L* values are the main indicators. As far as consumer acceptance is concerned, a higher L* value would be more desirable [24]. The study found that the L* value of the broiler breast muscle in the T2 group was significantly higher than that of the control group; the difference in the a* values of the broiler breast muscle across the test groups was not significant, while the b* value of the broiler breast muscle in the test groups was significantly lower than that of the control group. Suliman et al. [25] demonstrate that recognizing the color is a straightforward way of measuring the pH of the meat; for example, if the meat is dark (high L* value), the pH is high, with chicken meat with a high pH exhibiting a greater water-retention capacity than that of meat with a low pH. Therefore, we postulate that *Bacillus subtilis KG109* increases the L* value of meat color by increasing the meat’s water-holding capacity, hence improving broiler meat quality.

### 4.3. Effect of Dietary Supplementation with Bacillus subtilis KG109 on Serum Biochemical Indexes of Broiler Chickens

Serum biochemical indexes are essential to measure the nutritional metabolism and stress condition of the organism. ALT and AST play an important role in transamination, indicating the status of protein synthesis and catabolism [26,27]. The results of this experiment reveal that the serum ALT and AST activities of the experimental broiler groups were significantly greater than those of the control group, while the serum AST activity of the T3 broiler group was significantly higher than that of the T1 group. It suggests that the inclusion of *Bacillus subtilis* in the diet can increase the protein synthesis ability of the liver and maintain the body’s amino acid equilibrium.

Serum GLB and ALB are key markers of protein consumption and utilization [28] and are strongly connected with meat yield [29], with ALB additionally exhibiting pleiotropic properties such as antioxidant activity and modulation of immunological and inflammatory responses [30]. The results of this experiment show that, compared with the control group, the serum GLB in the T2 and T3 groups had a tendency to increase following *Bacillus subtilis* supplementation, although the difference was not significant, and the serum ALB content was highly significantly increased. Abdel-Moneim et al. [31] show that increasing the amount of *Bacillus subtilis* in broiler feeds results in a linear increase in serum ALB levels. This is consistent with the outcomes from the current research. It suggests that including *Bacillus subtilis KG109* into the diet can boost protein deposition capacity in broilers. As a result, it is speculated that the reduction in FCR and the increase in slaughter rate and antioxidant properties observed in this study may be closely related to the significant increase in ALB. In this study, ALB continuously increased as the added amount of *Bacillus subtilis* increased, although the effect of a larger dose on ALB requires further investigation.

ALP is considered to be a biomarker for other tissues (such as bone and kidney tissues) [32]; it also plays an important role in lipid metabolism [33]. The results of this experiment show that serum ALP activity was significantly increased in all test groups compared to the control group. It has been established that adding *Bacillus subtilis* to the feed can increase the serum ALP activity of broiler chickens and diminish the generation of uric acid, changing the nutritional metabolism of broilers [34]. As a result, adding *Bacillus subtilis KG109* to the diet can boost fat, liver, and bone metabolism, hence promoting overall body growth and development.

Serum TG and TC content can reflect an organism’s ability to digest fat. The results of this experiment indicate that, compared to the control group, the serum TG of broilers in each test group was significantly reduced, especially in the T3 group, where the levels were extremely significantly lower than those in the control group and T2 group, but the difference was not significant in relation to the serum TC content. Cai et al. [35] showed that the addition of *Bacillus subtilis* to broiler diets leads to a significant reduction in serum TG levels in broilers. This could be because probiotics influence the host’s metabolic processes, especially lipid metabolism, resulting in decreased TG levels in broilers [36]. This is consistent with the results of the present experiment. Thus, *Bacillus subtilis* improves broiler development performance through the promotion of lipid metabolism and the increase in fat deposition capability.

### 4.4. Effect of Dietary Supplementation with Bacillus subtilis KG109 on Serum Antioxidant and Immunological Indexes in Broilers

The strength of an organism’s antioxidant capability is intimately related to its overall health. T-AOC, GSH-Px, T-SOD activity, and MDA content are key indexes for assessing the liver’s antioxidant capability. The results of this investigation reveal that the serum T-AOC, GSH-Px, and T-SOD activities in broilers increased dramatically with the addition of *Bacillus subtilis KG109* powder to their diet, whereas serum MDA content decreased significantly. Xu et al. [37] found that adding 1.5 × 10^9^ CFU/kg of *Bacillus subtilis* to broiler diets improved the antioxidant capacity by increasing serum GSH-Px, SOD, and CAT activities, lowering MDA levels and removing excess ROS. This is consistent with the findings of the current study which indicate that adding *Bacillus subtilis KG109* to the ration prevents the oxidation of hydrogen peroxide, preventing oxidative damage to the cells, eliminating harmful substances from the biological metabolic process, and reducing biofilm damage.

The immunological response of animals is intimately tied to immunoglobulins, and it has been shown that serum immunoglobulins play a vital role in the immune function and in the resistance to many illnesses [38]. The results of this investigation demonstrate that adding *Bacillus subtilis KG109* to broiler diets significantly boosted serum IgG, IgM, and IgA levels. Qiu et al. [39] and Xu et al. [37] demonstrate that adding *Bacillus subtilis* to broiler diets significantly raises the concentration of immunoglobulins IgA, IgM, and IgG in the serum, hence improving broiler immunity. It has also been shown that the addition of *Bacillus subtilis* to mouse diets significantly increases the serum levels of IgG, IgM, and IgA in mice. This shows that the animals’ systemic immunological state, antibody generation, and ability to prevent and eliminate exogenous invading infections improve gradually after the addition of *Bacillus subtilis KG109*.

### 4.5. Effect of Dietary Supplementation with Bacillus subtilis KG109 on the Intestinal Morphology of the Duodenum and Jejunum of Broiler Chickens

VH, CD, and VCR are important indicators of the digestive and absorptive function, cell development, and maturation rate of the small intestine. The increase in small intestinal villi can strengthen the interface between the intestine and the nutrients and improve digestion and absorption. The greater the VCR, the higher the digestion and absorption capabilities [40]. The results of this study show that, compared with the control group, the differences in CD of the test groups over the control group were not significant, but the VH and VCR of the duodenum and jejunum were greatly enlarged. The intestinal morphology of the duodenum in the T2 group exhibited the best improvement, while the intestinal morphology of the jejunum in the T3 group exhibited the best improvement. This suggests that adding *Bacillus subtilis KG109* to chicken diets can improve intestinal mucosal structure, resulting in increased digestion and absorption. Sen et al. [41] and Dong et al. [42] found that dietary *Bacillus subtilis* significantly enhances VH and VCR in broiler chickens’ small intestines, but there was no significant difference in crypt depth. These findings are consistent with those from the current study. Additionally, higher VH and VCR are beneficial for broiler growth and intestinal food absorption [43], as intestinal villus lengthening contributes to a high absorptive surface area, which is thought to be a crucial component to foster development performance [44]. Thus, our findings imply that adding *Bacillus subtilis* to the food improves intestinal morphology, which, in turn, enhances broiler chicken developmental performance.

Digestive enzymes, which are largely found in the digestive juices, help breakdown food’s proteins, lipids, and carbohydrates, as well as helping breakdown big molecules into smaller ones that the body can absorb and use [45]. The higher activity of protease, amylase, and lipase enhances the digestion of proteins, starch, and lipids, thereby promoting the growth performance of the animal organism [11]. The results of this experiment show that the activity of protease, amylase, and lipase in the duodenum and jejunum of broiler chickens increased with the increase in the dose of *Bacillus subtilis KG109* powder added to the diet compared to the control group. Some studies have shown that the addition of *Bacillus subtilis* to broiler baseline diets greatly boosts protease and amylase activity in the small intestine [46,47]. *Bacillus subtilis* not only secretes more exogenous digestive enzymes [48], but it also promotes the production of large quantities of digestive enzymes in the digestive tract, compensating for endogenous enzyme vacancies in the organism and, thus, improving nutrient utilization in feeds and increasing endogenous enzyme production [49]. Although the addition of 1.8 × 10^9^ CFU/kg *Bacillus subtilis* produced the best results in this experiment, more research is necessary to determine how larger doses affect digestive enzymes. A higher digestive enzyme activity leads to a more complete food digestion, which is also beneficial for absorption in the small intestine [45]. It has also been demonstrated that an increase in VH is associated with an increase in digestive enzyme activity, improving nutrient digestibility [50] and, possibly, accounting for the significant decrease in FCR caused by the addition of *Bacillus subtilis KG109* to broiler diets in this experiment.

## 5. Conclusions

Under the conditions of this experiment, adding *Bacillus subtilis KG109* to broiler diets significantly reduces broiler FCR, increases broiler slaughtering rate and serum ALT and ALP activity, increases ALB content, decreases serum TG content, increases broiler serum antioxidant and immune ability, improves intestinal morphology, and increases intestinal digestive enzyme activity compared to the control group. From these results, it can be evinced that *Bacillus subtilis KG109* plays a crucial role in broiler chicken production and performance. Adding 1.8 × 10^9^ CFU/kg yields the best results.

## Figures and Tables

**Table 1:** Composition and nutrient levels of basal diets (air-drying basis) %.

Items	1 to 21 Days of Age	22 to 52 Days of Age
Ingredients		
Corn	49.64	58.11
Soybean meal	31.88	17.85
Corn gluten meal	5.00	5.00
Soybean oil	3.20	6.10
Breadcrumbs	3.00	
Flour	3.00	2.00
Nucleotide residue		1.00
Feather powder		1.00
Glutamate residue		1.50
Citric acid residue		3.00
Lys	0.54	0.87
Mineral meal	1.34	1.30
CaHPO_4_	0.93	0.80
Sodium humate		0.10
Montmorillonite	0.20	0.10
NaCl	0.27	0.27
Premix ^(1)^	1.00	1.00
Total	100.00	100.00
Nutrient level ^(2)^		
CP	21.55	19.15
EE	2.52	3.12
CF	3.80	4.45
Ash	6.93	7.38
ME/(MJ/Kg) ^(2)^	12.43	13.35
Met	0.55	0.46
Lys	1.13	1.00
Ca	1.15	1.04
TP	0.65	0.58

^(1)^ The premix provided the following per kg of basal diet in 1-to-21-day-old chicks: vitamin A 7500 IU, vitamin D_3_ 2250 IU, vitamin E 20 mg, vitamin B_2_ 6 mg, vitamin K_3_ 4 mg, vitamin B_12_ 0. 01 mg, vitamin B_6_ 3 mg, biotin 0.006 mg, folic acid 0.9 mg, choline 0.35 g, Cu 0.018 g, Mn 0.1 g, Fe 0.075 g, and Zn 0.1 g. The premix provided the following per kg of basal diet in 22-to-52-day-old chicks: vitamin A 7500 IU, vitamin D_3_ 1500 IU, vitamin E 15 mg, vitamin B_2_ 5.5 mg, vitamin K_3_ 3 mg, vitamin B_12_ 0.01 mg, vitamin B_6_ 3.6 mg, biotin 0.06 mg, folic acid 0.8 mg, choline 0.3 g, Cu 0.018 g, Mn 0.11 g, Fe 0.1 g, and Zn 0.1 g. ^(2)^ ME is a calculated value, while the others are measured.

**Table 2:** Effects of dietary *Bacillus subtilis KG109* on the growth performance of the broilers.

Items	CON	T1	T2	T3	*p*-Value
From 1 day to 21 days of age					
ADFI/g	28.95 ± 0.55	28.52 ± 0.57	28.54 ± 0.48	28.11 ± 0.53	0.744
ADG/g	18.57 ± 0.44	19.38 ± 0.47	18.94 ± 0.18	19.53 ± 0.29	0.275
F/G	1.56 ± 0.04 ^a^	1.48 ± 0.05 ^ab^	1.51 ± 0.03 ^ab^	1.44 ± 0.01 ^b^	0.014
From 22 days to 52 days of age					
ADFI/g	86.99 ± 2.13	85.57 ± 2.00	84.84 ± 1.97	85.46 ± 2.34	0.906
ADG/g	35.52 ± 1.09	36.83 ± 1.09	37.23 ± 1.14	38.00 ± 1.10	0.459
F/G	2.46 ± 0.05 ^a^	2.33 ± 0.03 ^ab^	2.29 ± 0.04 ^b^	2.26 ± 0.05 ^b^	0.002
From 1 day to 52 days of age					
ADFI/g	63.55 ± 1.47	62.53 ± 1.40	62.10 ± 1.31	62.30 ± 1.47	0.890
ADG/g	28.68 ± 0.68	29.78 ± 0.64	29.84 ± 0.67	30.54 ± 0.72	0.300
F/G	2.22 ± 0.03 ^a^	2.10 ± 0.02 ^b^	2.08 ± 0.03 ^b^	2.04 ± 0.04 ^b^	0.003

ADFI, average daily feed intake; ADG, average daily gain; FCR, feed conversion ratio; Con, control and basal diet; T1, basal diet + 6.0 × 10^8^ CFU/kg *Bacillus subtilis KG109*; T2, basal diet + 1.2 × 10^9^ CFU/kg *Bacillus subtilis KG109*; T3, basal diet + 1.8 × 10^9^ CFU/kg *Bacillus subtilis KG109*. Means within a row with different letters (a, b) are statistically significantly different (*p* < 0.05). The experimental results are expressed as the mean ± standard error of the mean (SEM).

**Table 3:** Effects of dietary *Bacillus subtilis KG109* on the slaughter performance of the broilers (%).

Items	CON	T1	T2	T3	*p*-Value
Slaughter rate	84.24 ± 1.08 ^b^	85.94 ± 0.35 ^ab^	86.57 ± 0.56 ^a^	86.82 ± 0.30 ^a^	0.004
Semi-eviscerated rate	79.10 ± 1.01	79.32 ± 0.88	81.28 ± 0.58	80.95 ± 0.66	0.144
Eviscerated rate	67.41 ± 0.49	67.72 ± 0.53	67.79 ± 0.54	68.65 ± 0.19	0.280
Breast muscle rate	20.67 ± 0.53	20.87 ± 0.45	21.29 ± 0.57	21.74 ± 0.39	0.365
Leg muscle rate	20.45 ± 0.46	20.71 ± 0.40	20.81 ± 0.43	20.61 ± 0.46	0.948
Abdominal fat rate	2.29 ± 0.23	1.75 ± 0.25	1.91 ± 0.30	2.01 ± 0.38	0.533

Con, control and basal diet; T1, basal diet + 6.0 × 10^8^ CFU/kg *Bacillus subtilis KG109*; T2, basal diet + 1.2 × 10^9^ CFU/kg *Bacillus subtilis KG109*; T3, basal diet +1.8 × 10^9^ CFU/kg *Bacillus subtilis KG109*. Means within a row with different letters (a, b) are statistically significantly different (*p* < 0.05). The experimental results are expressed as the mean ± standard error of the mean (SEM).

**Table 4:** Effects of dietary *Bacillus subtilis KG109* on the breast muscle quality of the broilers.

Items	CON	T1	T2	T3	*p*-Value
pH_1h_	5.68 ± 0.02	5.74 ± 0.04	5.76 ± 0.04	5.67 ± 0.04	0.228
Drip loss rate/%	2.39 ± 0.32	2.15 ± 0.13	2.15 ± 0.19	2.18 ± 0.15	0.828
Cooking loss/%	21.74 ± 0.87 ^a^	19.86 ± 0.41 ^b^	19.12 ± 0.59 ^b^	19.87 ± 0.48 ^ab^	0.003
Shear force/MPa	2.19 ± 0.13	2.10 ± 0.24	2.13 ± 0.22	1.92 ± 0.19	0.944
L*	48.27 ± 0.65 ^b^	50.55 ± 0.84 ^ab^	51.10 ± 1.00 ^a^	49.44 ± 0.76 ^ab^	0.042
a*	11.13 ± 0.85	9.81 ± 0.55	9.76 ± 0.72	10.95 ± 0.83	0.425
b*	19.56 ± 0.94 ^a^	15.91 ± 0.64 ^b^	16.46 ± 0.86 ^b^	15.44 ± 0.57 ^b^	0.002

L*, lightness; a*, redness; b*, yellowness; Con, control and basal diet; T1, basal diet + 6.0 × 10^8^ CFU/kg *Bacillus subtilis KG109*; T2, basal diet + 1.2 × 10^9^ CFU/kg *Bacillus subtilis KG109*; T3, basal diet +1.8 × 10^9^ CFU/kg *Bacillus subtilis KG109*. Means within a row with different letters (a, b) are statistically significantly different (*p* < 0.05). The experimental results are expressed as the mean ± standard error of the mean (SEM).

**Table 5:** Effects of dietary *Bacillus subtilis KG109* on the serum biochemical indexes of the broilers.

Items	CON	T1	T2	T3	*p*-Value
ALT/(U/L)	3.97 ± 0.53 ^b^	5.07 ± 0.54 ^a^	4.10 ± 0.32 ^a^	4.14 ± 0.43 ^a^	0.032
AST/(U/L)	271.70 ± 12.54 ^ab^	253.29 ± 5.28 ^b^	271.66 ± 7.68 ^ab^	282.37 ± 11.61 ^a^	0.022
ALP/(U/L)	1637.30 ± 176.30 ^b^	3059.18 ± 373.15 ^a^	3031.14 ± 360.64 ^a^	3024.13 ± 371.61 ^a^	0.010
TP/(g/L)	35.05 ± 0.78	35.23 ± 1.26	37.51 ± 1.24	36.83 ± 1.84	0.481
ALB/(g/L)	11.18 ± 0.19 ^b^	11.46 ± 0.33 ^b^	12.31 ± 0.25 ^a^	12.57 ± 0.25 ^a^	0.010
GLB/(g/L)	23.18 ± 0.63	22.90 ± 0.88	24.70 ± 1.04	24.26 ± 1.87	0.701
A/G(%)	0.47 ± 0.01	0.50 ± 0.03	0.51 ± 0.01	0.58 ± 0.11	0.633
UA/(mmol/L)	0.67 ± 0.04	0.56 ± 0.06	0.54 ± 0.04	0.56 ± 0.04	0.169
TC/(mmol/L)	3.09 ± 0.29	2.94 ± 0.11	3.31 ± 0.16	3.40 ± 0.14	0.342
TG/(mmol/L)	0.27 ± 0.02 ^a^	0.23 ± 0.02 ^ab^	0.26 ± 0.02 ^a^	0.19 ± 0.01 ^b^	0.002

ALT, alanine transaminase; AST, aspartate transaminase; ALP, alkaline phosphatase; TP, total protein; ALB, albumin; GLB, globulin; UA, uric acid; TC, total cholesterol; TG, triglycerides; Con, control and basal diet; T1, basal diet + 6.0 × 10^8^ CFU/kg *Bacillus subtilis KG109*; T2, basal diet + 1.2 × 10^9^ CFU/kg *Bacillus subtilis KG109*; T3, basal diet +1.8 × 10^9^ CFU/kg *Bacillus subtilis KG109*. Means within a row with different letters (a, b) are statistically significantly different (*p* < 0.05). The experimental results are expressed as the mean ± standard error of the mean (SEM).

**Table 6:** Effects of dietary *Bacillus subtilis KG109* on the serum antioxidant and immune indexes of the broilers.

Items	CON	T1	T2	T3	*p*-Value
T-SOD/(U/mL)	105.62 ± 1.43 ^d^	115.09 ± 2.56 ^c^	127.88 ± 1.80 ^b^	134.58 ± 1.21 ^a^	<0.001
GSH-Px/(U/L)	23.03 ± 0.56 ^d^	27.43 ± 0.73 ^c^	30.30 ± 0.37 ^b^	32.96 ± 0.80 ^a^	<0.001
T-AOC/(U/L)	86.92 ± 1.44 ^d^	107.87 ± 2.39 ^c^	116.32 ± 2.48 ^b^	123.45 ± 2.35 ^a^	<0.001
MDA/(nmol/mL)	13.63 ± 0.39 ^d^	13.08 ± 0.39 ^d^	10.78 ± 0.27 ^b^	8.46 ± 0.28 ^a^	<0.001
IgG/(μg/mL)	9.05 ± 0.19 ^d^	10.89 ± 0.29 ^c^	12.08 ± 0.30 ^b^	13.22 ± 0.33 ^a^	<0.001
IgM/(ng/mL)	342.18 ± 4.45 ^d^	379.17 ± 3.59 ^c^	400.07 ± 3.34 ^b^	421.58 ± 3.15 ^a^	<0.001
IgA/(ng/mL)	407.97 ± 3.85 ^d^	434.31 ± 3.75 ^c^	460.38 ± 5.19 ^b^	502.90 ± 5.48 ^a^	<0.001

T-SOD, total superoxide dismutase; T-AOC, total antioxidant capacity; MDA, malondialdehyde; GSH-Px, glutathione peroxidase; Con, control and basal diet; T1, basal diet + 6.0 × 10^8^ CFU/kg *Bacillus subtilis KG109*; T2, basal diet + 1.2 × 10^9^ CFU/kg *Bacillus subtilis KG109*; T3, basal diet +1.8 × 10^9^ CFU/kg *Bacillus subtilis KG109*. Means within a row with different letters (a, b, c, d) are statistically significantly different (*p* < 0.05). The experimental results are expressed as the mean ± standard error of the mean (SEM).

**Table 7:** Effect of dietary *Bacillus subtilis KG109* on the morphology of the duodenum and jejunum of the broilers.

Items	CON	T1	T2	T3	*p*-Value
Duodenum					
Villus height/μm	1410.02 ± 61.38 ^b^	1720.73 ± 59.91 ^a^	1865.59 ± 81.15 ^a^	1780.03 ± 63.76 ^a^	<0.001
Crypt depth/μm	278.96 ± 8.73	280.09 ± 9.29	264.98 ± 11.96	269.61 ± 10.99	0.699
VCR	5.08 ± 0.23 ^b^	6.18 ± 0.25 ^a^	7.14 ± 0.48 ^a^	6.70 ± 0.32 ^a^	0.001
Jejunum					
Villus height/μm	1177.15 ± 95.57 ^b^	1347.06 ± 39.93 ^b^	1434.39 ± 42.83 ^a^	1382.98 ± 68.74 ^a^	0.043
Crypt depth/μm	260.45 ± 12.55	230.91 ± 15.37	256.40 ± 17.97	223.07 ± 7.64	0.278
VCR	4.62 ± 0.44 ^b^	6.00 ± 0.35 ^a^	5.75 ± 0.37 ^a^	6.22 ± 0.28 ^a^	0.009

Con, control and basal diet; T1, basal diet + 6.0 × 10^8^ CFU/kg *Bacillus subtilis KG109*; T2, basal diet + 1.2 × 10^9^ CFU/kg *Bacillus subtilis KG109*; T3, basal diet +1.8 × 10^9^ CFU/kg *Bacillus subtilis KG109*. Means within a row with different letters (a, b) are statistically significantly different (*p* < 0.05). The experimental results are expressed as the mean ± standard error of the mean (SEM).

**Table 8:** Effect of dietary *Bacillus subtilis KG109* on digestive enzyme activity in the duodenum and jejunum of the broilers.

Items	CON	T1	T2	T3	*p*-Value
Duodenum					
Protease (U/mL)	15.57 ± 0.74 ^d^	20.55 ± 0.61 ^c^	24.80 ± 0.60 ^b^	28.53 ± 0.51 ^a^	<0.001
Amylase (U/L)	164.36 ± 4.00 ^d^	194.91 ± 3.83 ^c^	224.74 ± 4.18 ^b^	245.53 ± 3.84 ^a^	<0.001
Lipase (U/mL)	1.46 ± 0.09 ^d^	1.89 ± 0.06 ^c^	2.24 ± 0.05 ^b^	2.67 ± 0.06 ^a^	<0.001
Jejunum					
Protease (U/mL)	11.04 ± 0.48 ^d^	14.53 ± 0.58 ^c^	17.85 ± 0.47 ^b^	21.30 ± 0.57 ^a^	<0.001
Amylase (U/L)	116.16 ± 2.53 ^d^	130.02 ± 2.71 ^c^	151.72 ± 3.99 ^b^	174.88 ± 3.80 ^a^	<0.001
Lipase (U/mL)	0.95 ± 0.03 ^d^	1.08 ± 0.04 ^d^	1.39 ± 0.04 ^b^	1.71 ± 0.07 ^a^	<0.001

Means within a row with different letters (a, b, c, d) are statistically significantly different (*p* < 0.05). The experimental results are expressed as the mean ± standard error of the mean (SEM).

## Data Availability

The original contributions presented in the study are included in the article. Further inquiries can be directed to the corresponding author.

## Appendix A. Information on All the Kits Used to Measure Serum Biochemicals and Intestinal Digestive Enzymes in Broilers

IgA	IgA ELISA Kit	A045-4-1
IgM	IgM ELISA Kit	A110-1-1
IgG	IgG ELISA Kit	A110-1-1
T-SOD	Superoxide Dismutase (SOD) Assay Kit	A00-3-2
GSH/Px	Glutathione Peroxidase (GSH-Px) Kit	A005-1-1
T-AOC	Total Antioxidant Capacity (T-AOC) Kit	A015-2-1
MDA	Malondialdehyde (MDA) Kit	H003-1-2
Protease (U/mL)	Protease assay kit	W060-1-1
Lipase (U/L)	Lipase (LPS) assay kit	W054-1-1
Amylase (U/dL)	Total amylase (T-AMY) assay kit	C016-1-1
